# Father-inclusive chatbot-based prenatal education during COVID-19 pandemic enhances maternal–fetal attachment in Korean primigravida women across levels of partner support

**DOI:** 10.1038/s41598-025-13586-z

**Published:** 2025-08-05

**Authors:** Kyungmi chung, Kyungun Jhung, Hee Young Cho, Jin Young Park

**Affiliations:** 1 Institute of Behavioral Science in Medicine, Yonsei University College of Medicine, Yonsei University Health System, Seoul, Republic of Korea; 2 Department of Psychiatry, Yongin Severance Hospital, Yonsei University College of Medicine, Yonsei University Health System, Dongbaekjukjeon-daero 361, Giheung-gu, Yongin, 16995 Republic of Korea; 3 Center for Digital Health, Yongin Severance Hospital, Yonsei University College of Medicine, Yonsei University Health System, Yongin, Republic of Korea; 4 Department of Psychiatry, International St. Mary’s Hospital, Catholic Kwandong University College of Medicine, Incheon, Republic of Korea; 5 Department of Obstetrics and Gynecology, Seoul National University Hospital, Seoul National University College of Medicine, Daehak-ro 101, Jongno-gu, Seoul, 03080 Republic of Korea; 6 Institute of Reproductive Medicine and Population, Medical Research Center, Seoul National University, Seoul, Republic of Korea

**Keywords:** Patient education, Quality of life, Risk factors, Translational research

## Abstract

This study investigated whether a mobile chatbot-delivered intervention for prenatal education could enhance maternal-fetal attachment (MFA) among pregnant women with different levels of spousal support. In a quasi-experimental study conducted in January 2021, sixty volunteers who met all the eligibility criteria were enrolled: legally married, pregnant women aged 19 to 49 years, primigravida women from 21 to 32 weeks of gestation, and those in need of prenatal education. After completing a pre-questionnaire on pregnancy status, perception of prenatal education, and partner support, the women were asked to sequentially perform the given six tasks via a chatbot for two weeks, and then they filled out a post-questionnaire after completing the tasks. Both affective and cognitive-behavioral MFA outcomes were repeatedly measured before and after the intervention. The chatbot intervention enhanced affective MFA by increasing the intensity of prenatal education regardless of spousal support levels (*p* = .03): (1) low (*n* = 29; pre vs. post: *p* < .0042) and (2) high (*n* = 31; pre vs. post: *p* < .0042). In terms of cognitive-behavioral MFA, active paternal involvement in the chatbot-assisted baby talk helped women with low spousal support to differentiate themselves from the fetus (*p* = .02; pre vs. post: *p* < .0042) and to attribute characteristics and intention to the fetus (*p* = .011; pre vs. post: *p* < .0042), eliciting positive affect. This new mobile chatbot successfully served as a guide for pregnant couples starting prenatal education for the first time.

**Supplementary Information:**

The online version contains supplementary material available at 10.1038/s41598-025-13586-z.

## Introduction

### Background

 The presence of a supportive partner or spouse is crucial in mitigating challenges during the transition to parenthood, protecting maternal mental health^1^enhancing maternal-fetal attachment^2^and positively influencing pregnant women’s decision to seek prenatal care^3^. Pregnant women typically regard their husbands as their main source of perinatal support, followed by family members and friends, significantly influencing their happiness during pregnancy^4^. However, given the potential for negative dynamics within partner relationships, such as relationship breakdown or intimate partner violence, partner support may exhibit a dual nature, acting as both a risk and a protective factor. Antenatal depression or anxiety is strongly associated with perceived lack of partner and social support with informational (information and advice), instrumental (practical help), and emotional (expression of caring and holding in esteem) dimensions^5^. Conversely, perceived social support and marital satisfaction, active coping, and high self-esteem and self-efficacy can protect against antenatal depression or anxiety^6–8^. Antenatal mental health also significantly predicts postpartum depression along with poor marital relationship, stressful life events, negative attitude toward pregnancy, and inadequate social support^9^. In this respect, increased attention should be directed toward actively engaging male partners in perinatal education and providing tools to help men foster emotional bonds with their unborn child and effectively support their partners, thereby strengthening maternal well-being and facilitating a more inclusive, family-oriented perinatal care model.

Men preparing for fatherhood often feel uninformed and marginalized by formal, mother-focused perinatal resources, altered emotional connection with their partners, inflexible work arrangements, and traditional masculine norms^10–12^. This traditional, gendered view of parenthood, with mothers as primary caregivers and fathers as secondary supporters, has been consistently reported across diverse cultural contexts in North America, Europe, Asia, Oceania, and other regions worldwide^10–15^. As fathers prefer tailored information from informal, partner-inclusive interventions^16–18^female partners can serve as key information gateways^10,11^helping fathers feel well informed, involved, and prepared for perinatal care. Understanding fathers’ help-seeking behaviors and their specific support needs facilitates the development of father-friendly perinatal services by aligning men’s self-care with their family-oriented masculine ideals and roles as supporters, protectors, and co-parents, particularly through flexible, father-inclusive service platforms^10–15,19^. Primigravida women, having less childbirth knowledge and experience than multiparous women, rely heavily on external support from their spouses or professional services^20,21^. In addition, married pregnant women appear to receive more substantial partner support compared to unmarried women, both domestically and internationally^22,23^. According to the OECD Family Database (2023), the proportion of births among unmarried couples or single mothers was substantially lower in South Korea (2–3%) than in Europe (40–60%)^24^, suggesting legal marital status may influence societal acceptance and social support regarding pregnancy and childbirth. Furthermore, pregnant women’s recognition of prenatal education significantly enhances their active engagement and the effectiveness of educational interventions^25,26^. These findings underscore the unique vulnerability and greater receptivity of legally married first-time mothers with low spousal support to prenatal interventions involving fathers.

According to a systematic review and meta-analysis^27^prenatal attachment improves when interventions incorporate multiple attachment behaviors and skills provided via counselling and training programs. Educational intervention training first-time fathers in attachment skills, such as talking to the fetus while gently stroking the mother’s abdomen (i.e., ‘baby talk’ or ‘Taedam-taegyo’ in Korean), indirectly enhance maternal-fetal attachment (MFA) among their primigravida partners^28^. Compared with women educated alone or receiving no antenatal education, those participating in joint educational programs with their male partners demonstrated greater improvements in reproductive health knowledge and maternal health practices^29^highlighting benefits of couple-based interventions. A meta-analysis of psychoeducational programs for pregnant couples also revealed that these joint interventions improved postpartum mental health and relationship satisfaction by facilitating a healthy transition into parenthood^30^. In South Korea, prenatal care remains largely mother-centered due to traditional Confucian norms^31^. Prenatal education typically targets pregnant women in heterosexual relationships (64.4%)^32^ and is delivered face-to-face in small groups at hospitals or public health centers on fixed schedules (e.g., a single weekday or Saturday afternoon). These cultural, temporal, and spatial constraints limit partner-inclusive prenatal care^31,33^. Although traditional face-to-face prenatal interventions have demonstrated benefits for antenatal mental health and MFA in both maternal-only^23–26^ and partner-inclusive^20,21^ programs, the effectiveness of self-administered, mobile health (mHealth) prenatal interventions requiring paternal involvement has yet to be fully explored, particularly among pregnant women with different levels of partner support.

During the study period, the COVID-19 pandemic significantly disrupted prenatal education attendance in Korea and globally, with many in-person prenatal group sessions suspended or canceled due to infection and safety concerns. Pregnant women faced limited social support from family, friends, and even healthcare providers, resulting in increased reliance on their spouses as the primary support source^34^. In response, pregnant women increasingly preferred online prenatal education (54.4%) over traditional face-to-face methods (40.0%), primarily obtaining information from internet sources rather than healthcare providers. Nonetheless, comprehensive approaches combining knowledge transfer and practical training (77.8%) remained strongly preferred over single-method programs (11.1% each)^35^. Given these contextual challenges and shifts in educational preferences, chatbot-based prenatal education has emerged as a promising alternative. Interactive chatbots may effectively promote knowledge, positive health behaviors, and healthcare utilization among pregnant couples^36^. Particularly, chatbots are beneficial to deliver personalized, timely health information, filling gaps left by disrupted face-to-face interactions during the pandemic^37^. Furthermore, chatbots provide continuous, multidimensional support, increasingly appealing to digitally savvy parents^38^. However, little research has examined how chatbot-based prenatal education and spousal support affect MFA and maternal mental health during mid-pregnancy, when Korean pregnant women actively engage in prenatal care practices. Moreover, recent studies on digital prenatal education have not sufficiently explored whether mobile instant messaging chatbots effectively improve these outcomes, particularly as digital transformation of traditional prenatal education.

### Objectives

This study aims to evaluate the effects of a two-week mobile chatbot-based prenatal education program, designed to promote paternal involvement through both the chatbot agent and maternal encouragement, on maternal-fetal attachment (MFA) among pregnant women with different levels of perceived partner support. Based on the assumption that women with low partner support (low PS group) would show lower MFA than those with high partner support (high PS group), we hypothesized that (1) the intervention would enhance MFA in the low PS group to levels comparable to those in the high PS group; (2) active paternal involvement in the chatbot-assisted baby talk would contribute to greater relational satisfaction and emotional well-being across both groups.

## Methods

### Study design

For this study to be conducted in natural field settings, a quasi-experimental, two-group pretest–posttest design was adopted to evaluate the effectiveness of a mobile chatbot-based intervention on MFA in pregnant women with different levels of partner support. Partner support was dichotomized into low and high groups using a median split to clearly differentiate participants based on relative levels of perceived support, allowing for a direct examination of interaction effects between partner support groups and the intervention over time, thereby facilitating interpretation and practical application of the findings. There were two independent variables: partner support as a between-subjects factor and time as a within-subjects factor. In the 2 (partner support: low vs. high) $\:\times\:$ 2 (time: pretest vs. posttest) factorial design, two different concepts of MFA (i.e., cognitive-behavioral MFA and affective MFA) as a dependent variable, were repeatedly measured for primigravida women during mid-pregnancy (21–32 weeks of gestation)—once before and once after the intervention implementation. This study was approved by the institutional review board (IRB) of CHA Bundang Medical Center, CHA University. In accordance with the relevant guidelines and regulations of the IRB, all methods were performed, and electronic informed consent was obtained from all participants.

### Participant recruitment

To minimize the Hawthorne effect or the placebo effect by the doctor-patient relationship, pregnant women were recruited not from outpatient clinics to which our research team belongs but from a mobile research panel (https://www.panelnow.co.kr) of dataSpring Korea Inc., in January 2021. All screening and survey items were completed by pregnant women, regardless of whether their male partners had initiated contact in response to the study advertisement. Potential participants were screened for eligibility after obtaining electronic informed consent based on predefined inclusion and exclusion criteria. The inclusion criteria were (1) legally married, pregnant women aged 19 to 49 years, (2) primigravida women from 21 to 32 weeks of gestation, and (3) those who indicated a perceived need for prenatal education by selecting “neutral,” “somewhat needed,” or “absolutely needed” in response to the item: “To what extent do you perceive prenatal education as necessary as an expectant parent?” According to the exclusion criteria, (1) single or legally unmarried, pregnant women or pregnant widows aged under 19 years or over 49 years, (2) primigravida women at a gestation of < 21 weeks or > 32 weeks, (3) multigravida women, or (4) those who indicated no perceived need for prenatal education by selecting either “not needed at all” or “not needed” on the same item were excluded. Particularly, legal marital status was not verified through official documentation, but was self-reported by the women via the screening questionnaire. Considering the possibility that either legally married women or their male partners might have children from previous marriages or other prior relationships, women who reported living with children were excluded to control for the influence of prior birth experience. However, female participants living with extended family members such as their own parents or in-laws, who could provide additional sources of social support beyond the spouse, were included.

To control for potential confounders such as maternal age, marital status, and prior birth experience, rigorous eligibility criteria were established However, no specific age limits were placed on the male partners. Given Korea’s conservative, family-oriented Confucian culture, where premarital cohabitation is uncommon and marriage typically precedes childbirth, this study specifically targeted legally married, primigravida women to reflect normative patterns of prenatal partner support within the Korean sociocultural context. Consistent with this rationale, same-sex couples were excluded, as same-sex marriage is not legally recognized in Korea, and it was implicitly assumed that participating heterosexual couples were the biological parents of the fetus. Particularly for previous birth experience, participants who initially indicated having no previous birth experience (“no”) on the pre-questionnaire but later selected family structures explicitly including a child (i.e., “myself + husband + child,” “myself + husband + child + husband’s family,” or “myself + husband + child + my own family”) on the post-questionnaire were classified as providing inconsistent responses. To maximize participant engagement and receptivity, the sample was limited to married primigravida women who explicitly recognized the need for prenatal education.

In this study, participants who failed to meet any of the eligibility criteria were not enrolled; in addition, those who provided insincere or dubious responses to survey questions or withdrew from study participation were finally excluded from the data analysis. The research team identified these participants through visual inspection and response-pattern analysis, specifically excluding those who repeatedly selected identical responses across all items, exhibited obvious response patterns, or provided inconsistent answers to logically linked questions.

### Apparatus

**Fig. 1:**
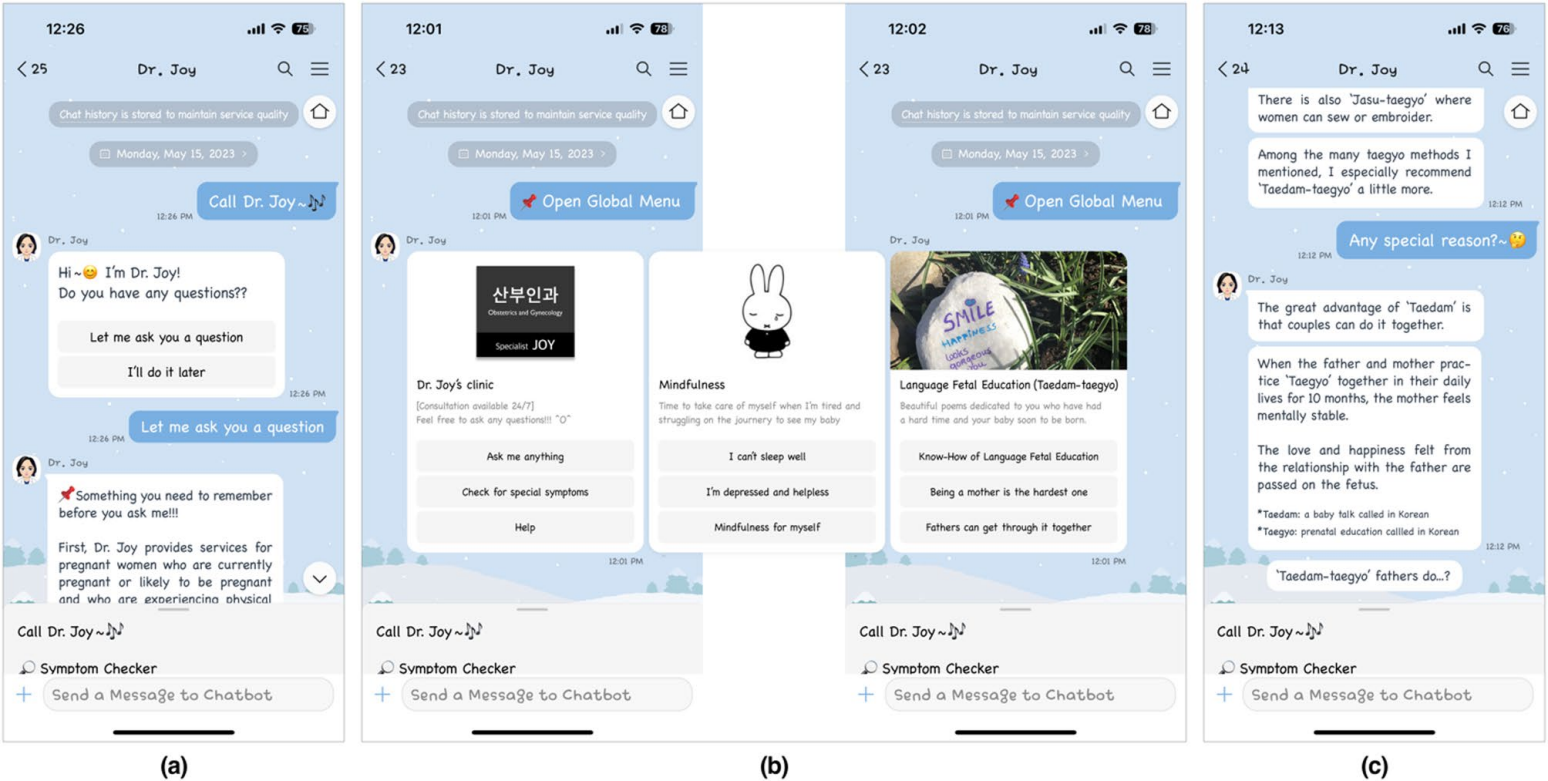
Screenshots of Dr. Joy’s (**a**) Q&A user interface, (**b**) three main menus for informational, instrumental, and emotional support, and (**c**) prenatal education contents for expectant fathers.

In our previous study based on perinatal couples’ unmet needs and barriers to in-home supportive services, we developed a medical chatbot (Dr. Joy) for perinatal women’s and their partners’ obstetric and mental health care, the content and services of which were available 24/7 via a mobile instant messaging app (i.e., KakaoTalk)^39^. Dr. Joy is a perinatal education tool geared toward promoting women’s self-care and their male partners’ engagement in maternity care, thus leading to (1) earlier psychoeducational intervention for women’s sleep problems and psychological distress among first-time pregnant couples and (2) strengthened MFA and partnership during pregnancy (Fig. 1 and Supplementary Fig. S1).

**Fig. 2:**
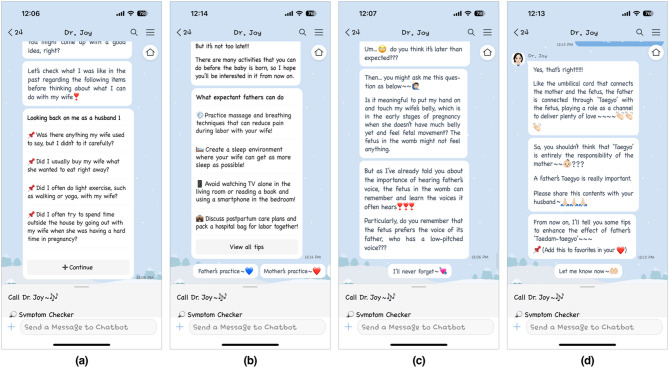
Screenshots of Dr. Joy’s detailed contents in father-inclusive prenatal education: (**a**) checklists for looking back on myself as a husband, (**b**) advice and tips for partner or spousal or support in everyday life, (**c**) comments for motivating fathers to participate in baby talk, and (**d**) recommendation for pregnant women to share the contents with their partners.

Dr. Joy was also designed with specific functionalities: (1) providing mothers with informal father-inclusive prenatal education and (2) encouraging them to share information with their male partners to be informed as suggested in the chatbot dialogue (Fig. 2 and Supplementary Fig. S2). Through the prenatal education program delivered by the chatbot, fathers can gain practical knowledge and tips on supporting their pregnant partners in daily life (e.g., reflecting on themselves and their past actions as social supporters, understanding the importance of participating in “prenatal education” (known as ‘Taegyo’ in Korean), and learning how to practice it). Fathers can also practice attachment behaviors and skills, initiating ‘Taedam-taegyo’ (baby talk in Korean) and interacting with both their partners and the unborn child (e.g., touching the belly, visualizing the fetal appearance, and talking to the fetus together at specified times) in a process-oriented manner via the chatbot.

### Measures

#### Support behaviors inventory (SBI)

To measure partner support, particularly spousal support in the Korean context, the shortened 11-item version of the Support Behaviors Inventory^40^ was adopted from the Prenatal Psychosocial Profile^41,42^. In this study, the Korean version of the SBI^43^ was administered to native Korean women. All married women were asked to indicate the degree to which they were satisfied with the support from their husbands on a 6-point scale ranging from 1 (very dissatisfied) to 6 (very satisfied); scores from the 11 items were all summed to yield a total SBI score ranging from 11 to 66, with higher scores indicating greater satisfaction with partner support.

#### Maternal fetal attachment scale (MFAS)

As a cognitive-behavioral MFA measure, the 24-item Maternal Fetal Attachment Scale^44^ was used to evaluate MFA defined as the “extent to which women engage in behaviors that represent an affiliation and interaction with their unborn child” (p. 282). Considering that both the course of pregnancy and fetus development and the transition to motherhood are major developmental periods for a pregnant woman, her fetus, and their relationship, we adopted the Korean version of the modified MFAS^45–47^. In line with Cranley (1981)’s theoretical constructs^44^the maternal-fetal relationship was identified through five subscales: (1) Differentiation of self from the fetus (DIFFSLF; 3 items), (2) Interaction with the fetus (INTERACT; 5 items), (3) Attributing characteristics and intention to the fetus (ATTRIBUT; 6 items), (4) Giving of self to nurture and protect the fetus (GIVINGSLF; 6 items), and (5) Role taking to embrace a new role as a mother (ROLETAK; 4 items). From the perspective of a cognitive-behavioral approach, MFAS components can be described as cognitive (DIFFSLF and ATTRIBUT) and behavioral (INTERACT, GIVINGSLF, and ROLETAK) in nature. Each item was rated on a 4-point scale from 1 (not at all) to 4 (all the time). The total MFAS score ranged from 24 to 96, with higher scores indicative of higher levels of enriching the MFA behaviors and cognitions toward the fetus.

#### Maternal antenatal attachment scale (MAAS)

As an affective MFA measure, the 19-item Maternal Antenatal Attachment Scale^48^ was utilized to assess MFA described as “the emotional tie or bond which normally develops between the pregnant woman and her unborn infant.”^49^ Compared to the MFAS, the MAAS focused specifically on the pregnant women’s thoughts and feelings about their developing fetus, differentiating the attitude toward the fetus from that toward the pregnancy state or the motherhood role. The MAAS consisted of two subscales: (1) Quality of involvement (11 items) and (2) Intensity (frequency) of preoccupation with the fetus (8 items). While ‘quality’ referred to the affective experiences (e.g., emotional closeness/distance, tenderness/irritation, positive/negative feelings, joyful/unpleasant anticipation, or a vivid/vague internalized representation of the fetus as a real person), ‘intensity’ referred to the amount of time spent in attachment mode (e.g., thinking about, talking to, or palpating the fetus)^48^. In this study, the Korean version of the MAAS^50^ was employed, participants were asked to rate each statement on a 5-point scale from 1 to 5. Following previous studies^48,51^we calculated scores of each MAAS subscale, (1) the quality score (10 items) ranging from 10 to 50, (2) the intensity score (8 items) ranging from 8 to 40, and (3) its total score (19 items) ranging from 19 to 95. Higher scores indicated higher levels of the maternal-fetal emotional bonding in terms of quality and intensity.

#### Impressions on mobile chatbot-based prenatal education program

To evaluate user experience with a mobile chatbot-delivered prenatal care service for pregnant couples, female participants were asked to answer an open-ended question about their thoughts and impressions of the participation in the chatbot-delivered prenatal education with their spouses, including the frequency of husband involvement in the 2-week prenatal education program via the mobile chatbot and the most impressive task completed. In particular, this engagement was directly related to specific chatbot-guided tasks, including regularly practicing “baby talk” with their female partners, applying daily prenatal care tips to support and care for them, and spending more joyful moments together. Given that this study intended to assess the chatbot-based prenatal education program specifically from the female participants’ perspective, it was not mandatory for spouses to provide responses; however, pregnant women were allowed to include their spouses’ responses to the questions when answering the survey themselves. All survey responses were exclusively reported by the female participants.

#### Procedure

Under an IRB-approved protocol, we applied the following procedure. Participants, who had sincerely completed all six tasks using the chatbot and both online pre- and post-questionnaires within the designated study period, received monetary compensation (worth 50,000 won) for their participation. To identify qualified participants, female volunteers, who submitted electronic informed consent online, were required to respond to screening questions in a pre-questionnaire. After completing the pre-questionnaire on basic demographics, pregnancy status, perception of prenatal education, partner support, and MFA at baseline (pre-intervention) to screen out ineligible applicants, enrolled participants performed the six tasks sequentially given via email and SMS for two weeks after the baseline assessment. As the final step, they were requested to fill out a post-questionnaire on MFA, as well as additional sociodemographic and pregnancy behavioral characteristics after completing all six tasks (post-intervention).

As one of the remarkable advantages of Dr. Joy-delivered prenatal education is that pregnant couples can practice it together, the purpose of this study and the importance of prenatal education with the father’s voice and engagement were first explained to female participants, who were then encouraged to carry out the given six tasks with their spouses during the study period, particularly for their unborn babies. If a new task was assigned before completing a previous one, participants were guided to complete the remaining tasks in order. All tasks were required to be performed for at least 10 min each. Three tasks were assigned in the first week and the other three in the second week.

The detailed six tasks were as follows: (1) registering the Dr. Joy channel on KakaoTalk and reviewing the main menu and its contents; (2) deciding on a baby nickname and then talking to the baby in the womb by calling his or her nickname every morning and evening; (3) reading aloud short texts or poems randomly presented by Dr. Joy and writing them down while thinking about the unborn baby; (4) encouraging the husband to touch or rub her tummy and read poems or fairy tales to the fetus using his own voice; (5) having a happy time together to deliver her feelings of happiness to the fetus; (6) sharing her thoughts and impressions on participating in the chatbot-delivered prenatal education with her spouse.

### Statistical analysis

Data analyses were performed with IBM SPSS Statistics for Windows (version 22). All hypotheses were tested at a two-sided significance level of 0.05. First, descriptive analyses were conducted using a chi-square test for categorical variables and an independent-samples *t*-test for continuous variables to ascertain whether the two PS groups were equivalent with respect to demographic characteristics. Second, a two-way mixed Repeated Measures Multivariate Analysis of Variance (RM-MANOVA) was performed to investigate the effects of group (low PS group vs. high PS group) and time (pre-intervention vs. post-intervention) on changes in two different concepts of cognitive-behavioral and affective MFAs. If a significant interaction was identified, Bonferroni-corrected planned pairwise comparisons were further performed (*p* <.05/12 = 0.0042).

## Results

### Participant characteristics

Sixty pregnant women, all of whom were first-time mothers, were aged 25 to 41 years (*M* = 31.92, *SD* = 3.03) with a gestational age of 21 to 32 weeks (*M* = 25.85, *SD* = 3.28). Based on a median split of their total SBI scores (median = 62.00), participants were divided into two subgroups: (1) low PS group (*n* = 29; SBI score < 62, range 11–61) and (2) high PS group (*n* = 31; SBI score ≥ 62, range 62–66). Participants’ demographic characteristics are presented in Table 1 in detail.

**Table 1:** Demographic characteristics of participants.

Characteristics	Total (*N* = 60)	Low PS^b^ group (*n* = 29)	High PS group (*n* = 31)	Test statistic	*p*-value
**Age (years)**				*t* (58) =.12^c^	0.91
*M* (*SD*)	31.92 (3.03)	31.97 (3.01)	31.87 (3.10)		
Range	25–41	25–37	27–41		
**Age groups**, ***n*** **(%)**				$\:{\chi\:}^{2}$ (2) = 1.84^d^	0.40
19–29 years	11 (18.3)	4 (13.8)	7 (22.6)		
30–39 years	48 (80.0)	25 (86.2)	23 (74.2)		
40–49 years	1 (1.7)	0 (0.0)	1 (3.2)		
**Education level**, ***n*** **(%)**				$\:{\chi\:}^{2}$ (3) = 5.00^d^	0.17
High school	2 (3.3)	2 (6.9)	0 (0.0)		
Technical college (2–3 years)	14 (23.3)	8 (27.6)	6 (19.4)		
Undergraduate degree (bachelor’s: 4–5 years)	38 (63.3)	18 (62.1)	20 (64.5)		
Postgraduate degree (master’s)	6 (10.0)	1 (3.4)	5 (16.1)		
**Current employment status of wife**, ***n*** **(%)**				$\:{\chi\:}^{2}$ (3) = 2.56^d^	0.46
Employed (full-time worker)	29 (48.3)	12 (41.4)	17 (54.8)		
Employed (part-time worker)	3 (5.0)	1 (3.4)	2 (6.5)		
On maternity leave	9 (15.0)	4 (13.8)	5 (16.1)		
Unemployed (housewife)	19 (31.7)	12 (41.4)	7 (22.6)		
**Current employment status of husband**, ***n*** **(%)**				$\:{\chi\:}^{2}$ (1) = 1.09^d^	0.30
Employed (full-time worker)	59 (98.3)	28 (96.6)	31 (100.0)		
On paternity leave	1 (1.7)	1 (3.4)	0 (0.0)		
**Gestational age**, ***n*** **(%)**				$\:{\chi\:}^{2}$ (1) =.65^d^	0.42
Second trimester (21–28 weeks)	47 (78.3)	24 (82.8)	23 (74.2)		
Third trimester (29–32 weeks)	13 (21.7)	5 (17.2)	8 (25.8)		
**Planned pregnancy**, ***n*** **(%)**				$\:{\chi\:}^{2}$ (1) =.20^d^	0.65
Yes	45 (75.0)	21 (72.4)	24 (77.4)		
No	15 (25.0)	8 (27.6)	7 (22.6)		
**Current smoking during pregnancy**, ***n*** **(%)**				$\:{\chi\:}^{2}$ (1) = 4.43^d^	0.04^*^
Non-smoker	53 (88.3)	23 (79.3)	30 (96.8)		
Ex-smoker (temporary abstinence during pregnancy)	7 (11.7)	6 (20.7)	1 (3.2)		
**Current alcohol drinking during pregnancy**, ***n*** **(%)**				$\:{\chi\:}^{2}$ (2) = 1.36^d^	0.51
Non-drinker	12 (20.0)	4 (13.8)	8 (25.8)		
Ex-drinker (temporary abstinence during pregnancy)	46 (76.7)	24 (82.8)	22 (71.0)		
Current drinker	2 (3.3)	1 (3.4)	1 (3.2)		
**Partner support (PS)**				*t* (31.35) = −9.54^e^	< 0.001^***^
*M* (*SD*)	59.88 (6.32)	54.76 (5.44)	64.68 (1.38)		
**Frequency of husband involvement in 2-week prenatal education via mobile chatbot** ^a^				*t* (11) = 14.64^c^	0.20
*M* (*SD*)	7.92 (4.18)	6.86 (3.46)	8.90 (4.59)		
Range	2–20	2–14	3–20		

^a^Except for this variable measured after intervention, all variables were measured at baseline. ^b^PS: Partner support; ^c^*t* (df) of independent samples *t*-test; ^d^$\:{\chi\:}^{2}$ (df) of chi-square test. ^e^Levene’s Test for Equality of Variances was not assumed. ^*^p <.05; ^**^p <.01; ^***^p <.001.

According to the results of chi-square tests and independent-samples *t*-tests shown in Table 1, the equivalence of the proportions and means between the two PS groups was confirmed (*p* >.05), indicating there was no significant difference between the groups in any of the reported demographic characteristics except for current smoking status. In terms of current maternal smoking during pregnancy, some women reported smoking cessation for their babies during pregnancy even if they were smokers before pregnancy; therefore, this indicated that there was no group difference in current smoking status. Most importantly, the pre–post treatment effects of the mobile chatbot-based intervention on MFA among pregnant women with different levels of partner support were not confounded by their spousal participation rate itself since no significant difference was observed between the groups regarding the frequency of husband involvement in the 2-week prenatal education via mobile chatbot.

**Table 2:** Descriptive statistics and ANOVA results for maternal-fetal attachment outcomes (MFAS and MAAS) by partner support group and measurement time.

Dependent variables	Time	Group, M (SD)		Group effect		Time effect		Interaction effect	
		Low PS group (*n* = 29)	High PS group (*n* = 31)	Test statistic	*p*-value	Test statistic	*p*-value	Test statistic	*p*-value
					$\:{\eta\:}_{p}^{2}$ ^c^		$\:{\eta\:}_{p}^{2}$		$\:{\eta\:}_{p}^{2}$
**MFAS**									
DIFFSLF	Pre^a^	10.24 (1.53)	11.32 (1.05)	*F* (1, 58) = 7.22	0.0009^**^	*F* (1, 58) = 7.96	0.007^**^	*F* (1, 58) = 5.47	0.02^*^
	Post^b^	10.93 (1.28)	11.39 (0.99)		0.11		0.12		0.09
INTERACT	Pre	13.41 (2.90)	15.19 (2.87)	*F* (1, 58) = 4.35	0.04^*^	*F* (1, 58) = 19.31	< 0.001^***^	*F* (1, 58) = 1.52	0.22
	Post	15.31 (2.59)	16.26 (3.00)		0.07		0.25		0.03
ATTRIBUT	Pre	17.31 (2.94)	20.19 (2.99)	*F* (1, 58) = 8.59	0.005^**^	*F* (1, 58) = 5.06	0.03^*^	*F* (1, 58) = 6.92	0.011^*^
	Post	18.97 (3.05)	20.06 (2.77)		0.13		0.08		0.11
GIVINGSLF	Pre	15.52 (3.16)	17.74 (3.90)	*F* (1, 58) = 5.33	0.02^*^	*F* (1, 58) = 4.27	0.04^*^	*F* (1, 58) = 0.72	0.40
	Post	16.52 (3.32)	18.16 (3.56)		0.08		0.07		0.01
ROLETAK	Pre	10.76 (3.20)	12.29 (2.77)	*F* (1, 58) = 4.19	0.045^*^	*F* (1, 58) = 7.88	0.007^**^	*F* (1, 58) = 0.26	0.61
	Post	11.55 (3.04)	12.84 (2.21)		0.07		0.12		0.005
Total MFAS score	Pre	67.24 (10.49)	76.74 (11.18)	*F* (1, 58) = 8.76	0.004^**^	*F* (1, 58) = 19.38	< 0.001^***^	*F* (1, 58) = 5.00	0.03^*^
	Post	73.28 (10.12)	78.71 (9.67)		0.13		0.25		0.08
**MAAS**									
Quality	Pre	31.41 (4.20)	35.16 (2.61)	*F* (1, 58) = 11.34	0.0014^**^	*F* (1, 58) = 12.88	0.0007^***^	*F* (1, 58) = 10.19	0.002^**^
	Post	33.62 (3.65)	35.29 (2.81)		0.16		0.18		0.15
Intensity	Pre	35.00 (4.39)	38.48 (4.16)	*F* (1, 58) = 7.36	0.009^**^	*F* (1, 58) = 40.85	< 0.001^***^	*F* (1, 58) = 4.79	0.03^*^
	Post	37.90 (4.14)	39.90 (3.82)		0.11		0.41		0.08
Total MAAS score	Pre	70.66 (8.30)	78.16 (6.71)	*F* (1, 58) = 10.19	0.002^**^	*F* (1, 58) = 32.84	< 0.001^***^	*F* (1, 58) = 9.81	0.003^**^
	Post	75.83 (7.56)	79.68 (6.34)		0.15		0.36		0.14

Note. MFAS: Maternal-Fetal Attachment Scale; DIFFSLF: Differentiation of self from the fetus; INTERACT: Interaction with the fetus; ATTRIBUT: Attributing characteristics and intention to the fetus; GIVINGSLF: Giving of self; ROLETAK: Role taking; MAAS: Maternal Antenatal Attachment Scale; PS: Partner support. Significant group $\:\times\:$ time interaction effects were found for DIFFSLF, ATTRIBUT, total MFAS, and all MAAS outcomes, indicating greater improvements in the low PS group. ^*^p <.05; ^**^p <.01; ^***^p <.001.

^a^Pre: Pre-intervention; ^b^Post: Post-intervention; ^c^$\:{\eta\:}_{p}^{2}$ = partial eta squared (effect size: small = 0.01, medium = 0.06, large ≥ 0.14).

### Effects of chatbot-based prenatal education and partner support level on maternal-fetal attachment

#### Cognitive-behavioral maternal-fetal attachment

**Fig. 3:**
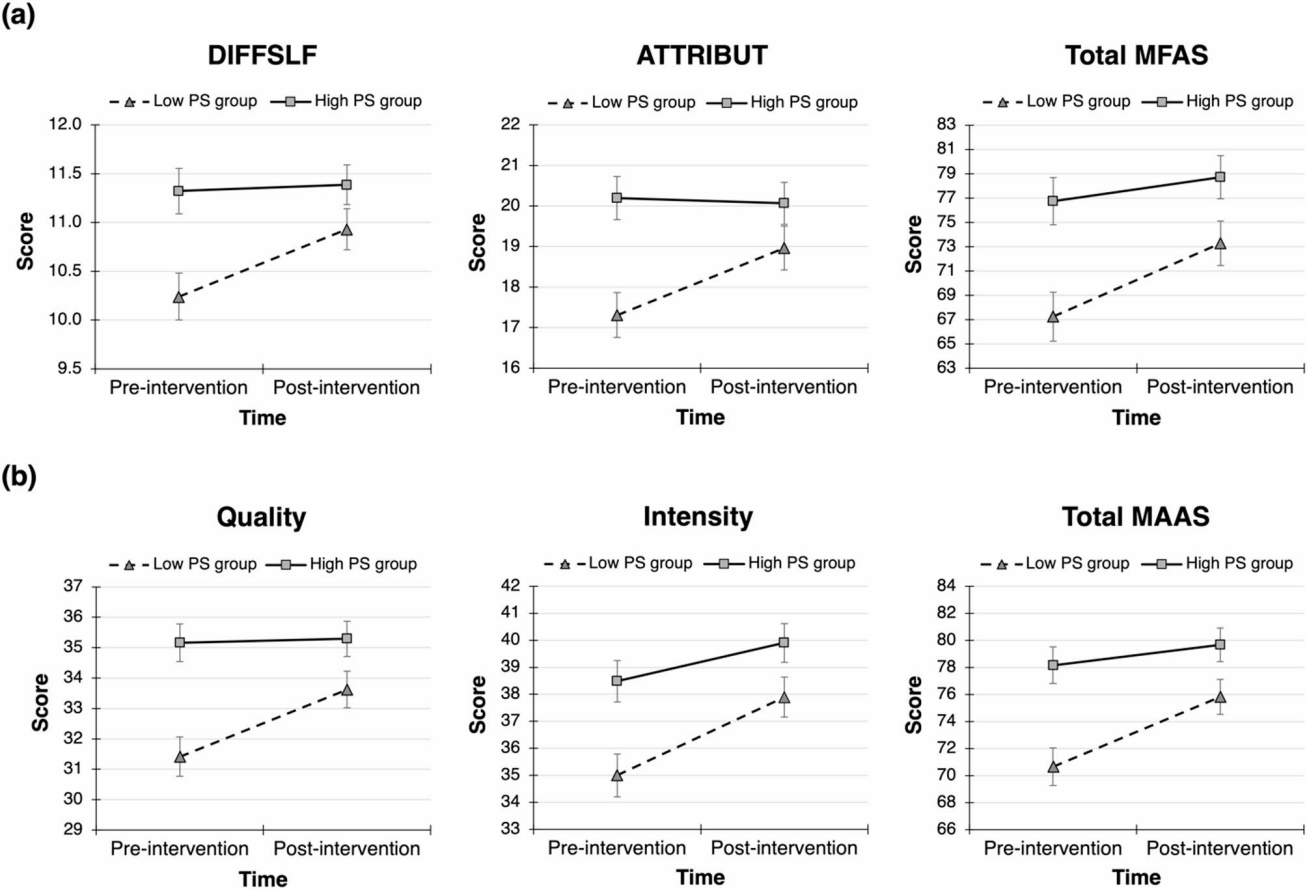
Plots of interaction effects of group and time on maternal-fetal attachment outcomes: (**a**) cognitive-behavioral maternal-fetal attachment (MFAS) and (**b**) affective maternal-fetal attachment (MAAS). MFAS: Maternal-Fetal Attachment Scale; DIFFSLF: Differentiation of self from the fetus; ATTRIBUT: Attributing characteristics and intention to the fetus; MAAS: Maternal Antenatal Attachment Scale; PS: Partner support. Error bars represent standard errors (SEs). Significant group $\:\times\:$ time interactions indicate greater improvements in the maternal-fetal attachment outcomes for the low PS group compared to the high PS group.

Regarding the MFAS, the 2 $\:\times\:$ 2 mixed RM-MANOVA tests on six outcomes (i.e., DIFFSLF, INTERACT, ATTRIBUT, GIVINGSLF, ROLETAK, and total MFAS scores) were performed between the two PS groups who had the same intervention for two weeks. The multivariate tests on the MFAS showed a significant main effect of time [Wilks’ Lambda ($\:\lambda\:$) = 0.68, *F* (5, 54) = 5.10, *p* <.001, partial eta squared ($\:{\eta\:}^{2}$) = 0.32]; however, no significant main effect of group (Wilks’ $\:\lambda\:$ = 0.84, *F* (5, 54) = 2.07, *p* =.08, partial $\:{\eta\:}^{2}$ = 0.16) or group $\:\times\:$ time interaction [Wilks’ $\:\lambda\:$ = 0.85, *F* (5, 54) = 1.92, *p* =.11, partial $\:{\eta\:}^{2}$ = 0.15] was found. As shown in Table 2, the subsequent univariate tests revealed that the main effects of group and time were all significant for the six outcomes (all *p* <.05). Except for INTERACT, GIVINGSLF, and ROLETAK, the interaction effects between group and time were significant for the other three outcomes (all *p* <.05, see Table 2; Fig. 3). The significant interaction effects observed for the MFAS outcomes showed medium effect sizes, as indicated by partial $\:{\eta\:}^{2}$ values ranging from 0.08 to 0.11.

According to the results of planned post-hoc pairwise comparisons (see Table 3), the low PS group showed a significant increase in the three MFAS outcomes (i.e., DIFFSLF, ATTRIBUT, and total MFAS score) after the 2-week chatbot-delivered intervention (all *p* <.0042). In contrast, the high PS group showed no significant difference in the same outcomes after the intervention (all *p* >.0042), indicating that partner support was sufficient to maintain the high levels of MFA regardless of the intervention. As we hypothesized, the low PS group showed lower MFA, indexed by DIFFSLF, ATTRIBUT, and total MFAS score, than the high PS group at baseline (all *p* <.0042). Following the mobile chatbot-based intervention, no significant group difference in the three MFAS outcomes was found after 2 weeks (all *p* >.0042).

**Table 3:** Planned pairwise comparison for significant group $\:\times\:$ time interaction effects on maternal-fetal attachment outcomes (MFAS and MAAS).

Dependent variables	Pairwise comparison	Mean difference	SE	Test statistic	*p*-value^d^
	Group $\:\times\:$ Time vs. Group $\:\times\:$ Time				
**MFAS**					
DIFFSLF	Low PS $\:\times\:$ Pre^a^ – Low PS $\:\times\:$ Post^b^	− 0.69	0.22	*t* (28) = −3.18	0.0036^*^
	High PS $\:\times\:$ Pre – High PS $\:\times\:$ Post	− 0.06	0.16	*t* (30) = − 0.40	0.69
	Low PS $\:\times\:$ Pre – High PS $\:\times\:$ Pre	−1.08	0.34	*t* (49.11) = −3.18^c^	0.0026^*^
	Low PS $\:\times\:$ Post – High PS $\:\times\:$ Post	− 0.46	0.29	*t* (58) = −1.55	0.13
ATTRIBUT	Low PS $\:\times\:$ Pre – Low PS $\:\times\:$ Post	−1.66	0.48	*t* (28) = −3.42	0.0020^*^
	High PS $\:\times\:$ Pre – High PS $\:\times\:$ Post	0.13	0.47	*t* (30) = 0.27	0.79
	Low PS $\:\times\:$ Pre – High PS $\:\times\:$ Pre	−2.88	0.77	*t* (58) = −3.76	0.0004^*^
	Low PS $\:\times\:$ Post – High PS $\:\times\:$ Post	−1.10	0.75	*t* (58) = −1.46	0.15
Total MFAS score	Low PS $\:\times\:$ Pre – Low PS $\:\times\:$ Post	−6.03	1.46	*t* (28) = −4.12	0.0003^*^
	High PS $\:\times\:$ Pre – High PS $\:\times\:$ Post	−1.97	1.10	*t* (30) = −1.78	0.08
	Low PS $\:\times\:$ Pre – High PS $\:\times\:$ Pre	−9.50	2.80	*t* (58) = −3.39	0.0013^*^
	Low PS $\:\times\:$ Post – High PS $\:\times\:$ Post	−5.43	2.55	*t* (58) = −2.13	0.04
**MAAS**					
Quality	Low PS $\:\times\:$ Pre – Low PS $\:\times\:$ Post	−2.21	0.55	*t* (28) = −4.00	0.0004^*^
	High PS $\:\times\:$ Pre – High PS $\:\times\:$ Post	− 0.13	0.36	*t* (30) = − 0.36	0.72
	Low PS $\:\times\:$ Pre – High PS $\:\times\:$ Pre	−3.75	0.90	*t* (58) = −4.18	0.0001^*^
	Low PS $\:\times\:$ Post – High PS $\:\times\:$ Post	−1.67	0.84	*t* (58) = −1.99	0.05
Intensity	Low PS $\:\times\:$ Pre – Low PS $\:\times\:$ Post	−2.90	0.52	*t* (28) = −5.58	0.00001^*^
	High PS $\:\times\:$ Pre – High PS $\:\times\:$ Post	−1.42	0.44	*t* (30) = −3.25	0.0029^*^
	Low PS $\:\times\:$ Pre – High PS $\:\times\:$ Pre	−3.48	1.10	*t* (58) = −3.15	0.0025^*^
	Low PS $\:\times\:$ Post – High PS $\:\times\:$ Post	−2.01	1.03	*t* (58) = −1.95	0.06
Total MAAS score	Low PS $\:\times\:$ Pre – Low PS $\:\times\:$ Post	−5.17	1.00	*t* (28) = −5.15	0.00002^*^
	High PS $\:\times\:$ Pre – High PS $\:\times\:$ Post	−1.52	0.63	*t* (30) = −2.42	0.02
	Low PS $\:\times\:$ Pre – High PS $\:\times\:$ Pre	−7.51	1.94	*t* (58) = −3.86	0.0003^*^
	Low PS $\:\times\:$ Post – High PS $\:\times\:$ Post	−3.85	1.80	*t* (58) = −2.14	0.04

Note. MFAS: Maternal-Fetal Attachment Scale; DIFFSLF: Differentiation of self from the fetus; ATTRIBUT: Attributing characteristics and intention to the fetus; MAAS: Maternal Antenatal Attachment Scale; PS: Partner support. Post-hoc pairwise comparisons revealed significant improvements across all maternal-fetal outcomes for the low PS group. In contrast, the high PS group showed significant improvement only in ‘Intensity’ subscale of the MAAS. ^a^Pre: Pre-intervention; ^b^Post: Post-intervention. ^c^Levene’s Test for Equality of Variances was not assumed. ^d^Bonferroni-corrected *p*-value of 0.0042 (0.05/12 pairs for the MFAS and the MAAS subscales each) was applied for multiple comparisons.

### Affective maternal-fetal attachment

For the analysis of the MAAS, the 2 $\:\times\:$ 2 mixed RM-MANOVA was also performed. According to the results of the multivariate tests on three outcomes (i.e., quality, intensity, and total MAAS scores), the main effects of group [Wilks’ $\:\lambda\:$ = 0.84, *F* (3, 56) = 3.67, *p* =.02, partial $\:{\eta\:}^{2}$ = 0.16] and time [Wilks’ $\:\lambda\:$ = 0.54, *F* (3, 56) = 15.94, *p* <.001, partial $\:{\eta\:}^{2}$ = 0.46], and the group $\:\times\:$ time interaction [Wilks’ $\:\lambda\:$ = 0.82, *F* (3, 56) = 4.11, *p* =.011, partial $\:{\eta\:}^{2}$ = 0.18] were all significant. Subsequent univariate tests showed significant main effects of group and time, as well as significant interaction effects (Fig. 3), for all three outcomes (all *p* <.05, see Table 2). For the MAAS outcomes, partial $\:{\eta\:}^{2}$ values for the significant interaction effects ranged from 0.08 to 0.15, indicating large effect sizes.

Based on the results of the planned pairwise comparisons on each subscale of the MAAS (all *p* <.0042, see Table 3), the low PS group also showed the significant increase in all three MAAS outcomes (i.e., quality, intensity, and total MAAS score) after the 2-week intervention. In contrast, only intensity among the MAAS outcomes resulted in a significant difference over the intervention period the high PS group (*p* <.0042), indicative of effective treatment for pregnant women with high PS levels. As hypothesized, the low PS group also showed lower MFA, indicated by quality, intensity, and total MAAS score, than the high PS group at baseline (all *p* <.0042). Consistent with the results for the three MFAS outcomes, the two PS groups showed no significant difference in the three MAAS outcomes after 2 weeks (all *p* >.0042).

## Discussion

The purpose of this study was to evaluate whether a two-week mobile chatbot-based education program designed to encourage paternal involvement could improve maternal-fetal attachment (MFA) among first-time pregnant women with different levels of perceived partner support (PS): low and high PS groups. To our knowledge, this is the first study to investigate both cognitive-behavioral and affective MFA outcomes in relation to spousal support within a mobile chatbot-based prenatal education program. Maternal-fetal attachment, conceptualized as the emotional bond that mothers develop towards their unborn babies, is typically measured during the second or third trimester of pregnancy when maternal awareness and interaction with the fetus become salient^29,44,49^. Particularly, improvement in prenatal MFA may be effectively achieved by expectant fathers’ participation in prenatal care and their training in attachment skills^28,29^even within Korea’s traditionally Confucian context emphasizing maternal roles and responsibilities throughout pregnancy and parenting. Given the predictive value of prenatal MFA for postnatal maternal and infant outcomes such as maternal mental health and mother-infant bonding^9^the present study hypothesized that father-inclusive prenatal care would effectively enhance prenatal MFA among primigravida women in the low PS group, whose postnatal outcomes were anticipated to be poorer, bringing their MFA levels up to those observed in the high PS group. Additionally, even among women already benefiting from high spousal support, the chatbot-based intervention was expected to further increase their engagement in prenatal bonding behaviors in daily life.

As a promising new therapeutic approach, Dr. Joy with a persona of a medical doctor specializing in obstetrics and gynecology was developed to (1) recommend active paternal involvement in prenatal education in the daily life context, (2) suggest contents for a father-friendly antenatal care service, and (3) encourage the women to share the contents with their spouses so that the pregnant couples come to practice attachment skills and behaviors together from the early stages of pregnancy. Importantly, this study was carried out during the prolonged COVID-19 pandemic. Within traditional Korean culture that highly values prenatal education, pandemic-related restrictions effectively shifted first-time pregnant women’s attention from face-to-face antenatal classes to mobile health (mHealth) services. Considering that paternal support in traditional Korean culture is largely limited to financial support driven by deeply rooted Confucian gender norms, our study specifically aimed to create opportunities for fathers to provide broader dimensions of social support to their pregnant partners. In this context, the present study adds to the literature on the feasibility of a mobile chatbot-delivered prenatal care service to provide three dimensions of social support (i.e., informational, instrumental, and emotional support) for pregnant couples.

### Principal findings

Among the six Maternal-Fetal Attachment Scale (MFAS) outcomes measuring cognitive-behavioral MFA, our hypotheses were partially supported for “differentiation of self from the fetus,” “attributing characteristics and intention to the fetus,” and total MFAS scores, reflecting enhanced maternal engagement behaviors such as affiliation and interaction with the fetus, particularly in the low PS group. By contrast, the remaining three cognitive-behavioral MFA subfactors of “interaction with the fetus,” “giving of self to nurture and protect the fetus,” and “role taking to embrace a new role as a mother” did not show significant changes, possibly because these subfactors may be more intrinsic to maternal identity development and less modifiable by external support, especially over a short intervention period. Since all female participants perceived a need for prenatal education, it is likely that certain MFA subfactors were already elevated at baseline. This self-selection effect may have constrained the extent to which the intervention could further enhance these subfactors or may have limited significant changes to only specific MFA subfactors. As baseline partner engagement (e.g., fathers’ initial involvement in prenatal bonding activities) was not measured, women with initially lower partner engagement may have had greater potential to benefit from the intervention. Furthermore, this predisposition toward maternal commitment may be culturally reinforced by traditional Confucian gender norms in Korean society, which emphasize maternal self-sacrifice and devotion during pregnancy. Accordingly, MFA subfactors such as “interaction with the fetus,” “giving of self,” and “role taking” may have already been internalized through culturally shaped maternal identity, thereby limiting the additional influence of the intervention in these domains.

To further explain these findings, the observed improvements in “differentiation of self from the fetus” and “attributing characteristics and intention to the fetus” might be explained by the process of fetal personification, as illustrated in participants’ open-ended responses (Supplementary Data S1, quotes 1–4). If the fathers’ active participation in the chatbot-guided baby talk with their voices elicited novel fetal responses not typically observed in the maternal-fetal dyad, the mothers could perceive the fetus as an independent entity with distinct, identifiable characteristics, rather than merely an extension of themselves. As the origin of attachment has been conceptualized as a process of ‘cue sensitivity’ wherein mothers can learn to recognize their babies’ characteristics through sensed cues (e.g., touch, voices, emotions, etc.) during pregnancy, perceiving the unborn baby as a distinct individual within the maternal body could also foster feelings of possessiveness and protectiveness^52–54^. For men in the role of fathers, bonding with the unborn baby appeared to strengthen their motivation to protect and care for their pregnant partners in this study. Building on the previous finding that pregnant couples observed increased fetal movements such as abdominal stroking, limb extension, and activity changes in response to specific voices^54^our study further suggests that consistent exposure to a low-pitched male voice, which may induce quickening, could help couples infer the baby’s preferences or recognition of the father’s voice (Supplementary Data S1, quotes 5 and 6). Importantly, the absence of significant improvement in “interaction with the fetus” among the low PS group does not necessarily indicate a lack of intervention effect. Instead, it may reflect how pregnant women interpreted their partners’ involvement not as a means of enhancing a direct connection with the fetus, but as a moment of emotional closeness with their partners or a shift in the couple dynamic. In this sense, the intervention may have exerted its influence not through increased maternal-fetal interaction per se, but indirectly by enhancing partner bonding or prompting a reappraisal of the partner’s role, particularly among those with limited initial partner support.

Both PS groups showed improved intensity of affective MFA, indicating the effectiveness of the mobile chatbot in increasing the frequency (i.e., intensity) of prenatal education for first-time expectant couples with varying levels of spousal support. With respect to the three MAAS outcomes of quality, intensity, and total MAAS scores, all hypotheses were accepted only in the low PS group, demonstrating strengthened maternal emotional bonding towards the fetus. Unlike the low PS group, the high PS group, potentially benefiting from quality spousal support since early pregnancy, showed significant improvement only in ‘intensity.’ This indicates that the combination of spousal involvement and chatbot intervention effectively encouraged this group to devote more time to digital prenatal education, practicing attachment behaviors and skills as recommended by Dr. Joy, with a set routine (twice daily, morning and night). Besides the scheduled time and location, participants engaged in prenatal education through the app, benefiting from its easy accessibility and interactivity at their convenience (Supplementary Data S1, quotes 7–11). Surprisingly, not only men but also women themselves recognized the pregnant woman as the subject of action for prenatal education, believing that traditional prenatal education programs were excessively formal for completion in their busy daily lives. After completing the tasks, changes in their perception of prenatal education were observed. In line with previous studies^10,11^women could act as a gateway to information and lower their men’s psychological barriers to mother-baby-oriented prenatal care, which is attributed to the chatbot service. Consistent with other related works with different service platforms^10–15,19^this newly developed father-friendly perinatal service on a mobile chatbot platform led fathers to faithfully perform their roles as a supporter and a co-parent while maintaining their masculine identity as a man within the program (Supplementary Data S1, quotes 12–14).

Last but not least, evidence for emotional support from the chatbot intervention was observed in the open-ended responses to the most impressive task completed. Pregnant women who mentioned the experience of prenatal education with their husbands accounted for 65% (39/60), whereas 35% (21/60) did not mention any spousal support, suggesting that at least one-third of the female participants may have felt disappointed or frustrated due to the lack of noticeable change in spousal support during the short-term intervention. Despite the women’s dissatisfaction with their husbands’ passive attitude toward the practical components of prenatal education such as engaging in learned attachment-related interactive activities, the potential of the chatbot intervention to facilitate fathers’ behavioral change was also evidenced by the men’s positive responses to their unfamiliar yet precious experience with the baby talk (Supplementary Data S1, quotes 15 and 16). According to the women’s retrospective self-reports (Supplementary Data S1, quotes 17–19), the sense of happiness and relaxation of bodily tension primarily stemmed from their husbands’ engagement in interacting with the fetus, thus extending the emotional experience of happiness from the pregnant women to the family as a whole, embracing their unborn baby through the couples’ shared engagement. Moreover, some women also reported experiencing complementary emotional support through conversations with the human-like chatbot agent (Supplementary Data S1, quotes 17–19). These findings suggest that the Dr. Joy chatbot successfully supported pregnant women’s psychological adaptation by validating emotional struggles during the perinatal period, promoting bodily and emotional relaxation, and enhancing relational connectedness through shared prenatal experiences with their partners and unborn child.

In light of these findings, prior studies using digital platform-based interventions for father-inclusive prenatal education have demonstrated considerable variability in participants’ characteristics, sociocultural backgrounds, methodological frameworks (e.g., target samples, intervention duration and frequency, educational content and prenatal activities), and measured attachment outcomes^38,55,56^. Most previous studies focused primarily on evaluating the general effectiveness of interventions based on partner involvement without considering baseline levels of perceived partner support, and few examined MFA at the subfactor level or integrated both affective and cognitive-behavioral domains simultaneously. The current findings align with existing evidence suggesting that enhanced mindful awareness of present-moment experiences, encompassing cognition, emotion, and bodily sensations, contributes to improved cognitive-behavioral MFA, specifically in the differentiation of self from the fetus and the attribution of characteristics and intention to the fetus, among Korean pregnant women sharing a common sociocultural context^57^. Although the findings from this study require careful interpretation due to the cultural specificity inherent in Korea, characterized by deeply embedded traditional gender roles, the observed benefits of paternal involvement in prenatal interventions, such as enhanced MFA, maternal mental health, and maternal bonding, are consistent with existing global research^17,28,30,31,58,59^. Furthermore, this study extends previous literature by demonstrating that the well-established benefits of paternal involvement from face-to-face prenatal education can be effectively delivered through a chatbot-based intervention integrated into KakaoTalk, the most widely used instant messaging app in Korea, highlighting the potential of digital health interventions to broaden access to prenatal support, particularly in remote or underserved areas, high-risk pregnancies, and emergency situations (e.g., pandemics or natural disasters).

### Limitations and future directions

This study has several limitations. While providing higher external validity, the quasi-experimental design lacks the rigor of randomized controlled trials, making the findings susceptible to selection bias and internal validity concerns. Despite using a two-group pretest–posttest design, random assignment was not employed, potentially leading to initial differences in measured and unmeasured variables and possible placebo effects. Additionally, the short intervention duration and the absence of follow-up data limit the evaluation of long-term impacts on MFA, maternal mental health, and even birth outcomes. Future studies should employ longer intervention periods with follow-up assessments to explore whether the observed improvements in MFA persist over time and translate into positive psychosocial outcomes such as sustained partner support, reduced postpartum depression, enhanced postnatal maternal and paternal attachment, and increased parenting confidence. Moreover, the study did not assess baseline paternal involvement in prenatal activities, which may have influenced individual variations in MFA outcomes. Given that all pregnant women expressed a perceived need for prenatal education, certain MFA subcomponents might have been elevated at baseline, potentially limiting the scope for further improvement. Future studies should incorporate measures of initial paternal involvement or evaluate changes in perceived partner support before and after the intervention to elucidate these dynamics.

Other limitations include the absence of demographic and clinical variables such as paternal characteristics (age, smoking status, alcohol consumption, etc.), pregnancy risk factors (elderly primigravida, multiple gestation, complications, etc.), and conception methods (natual pregnancy, intrauterine insemination, or in vitro fertilization), despite their known impact on maternal prenatal and postpartum outcomes. Therefore, these potential confounders should be comprehensively measured and controlled in future studies. Furthermore, paternal engagement with the chatbot intervention was evaluated exclusively through maternal self-reports, potentially introducing subjective bias. Future research should incorporate objective measures or direct paternal reports to provide a more accurate assessment of paternal involvement. Lastly, the generalizability of our findings is limited by traditional Confucian gender norms in Korea. Future research should examine diverse cultural contexts, utilize ecological momentary assessments to directly explore paternal-fetal attachment behaviors, and include clinical or birth outcomes to enhance the applicability and practical relevance of the results.

## Conclusions

In conclusion, the newly developed chatbot successfully supported pregnant couples starting prenatal education for the first time. This mobile chatbot-delivered intervention effectively enhanced the MFA of pregnant women with low partner support, increasing their MFA to levels similar to those with high partner support after the intervention. To further enhance the effect of this chatbot-based intervention involving spouses, prerequisites for the main program and the supplementary programs after the completion of all curricula should be considered in prenatal education targeting couples with low partner support.

## Electronic supplementary material

Below is the link to the electronic supplementary material.

Supplementary Material 1

## Data Availability

The datasets generated and analyzed during the current study are not publicly available due to the IRB-approved participant informed consent form that permits data sharing and transferring to designated third parties, but are available from the corresponding authors on reasonable request.

## Abbreviations

MFA: Maternal-fetal attachment
mHealth: mobile health
SBI: Support Behaviors Inventory
MFAS: Maternal Fetal Attachment Scale
DIFFSLF: Differentiation of self from the fetus
INTERACT: Interaction with the fetus
ATTRIBUT: Attributing characteristics and intention to the fetus
GIVINGSLF: Giving of self to nurture and protect the fetus
ROLETAK: Role taking to embrace a new role as a mother
MAAS: Maternal Antenatal Attachment Scale
PS: Partner support
RM-MANOVA: Repeated measures multivariate analysis of variance
