# Supplementary material for: Father-inclusive chatbot-based prenatal education during COVID-19 pandemic enhances maternal–fetal attachment in Korean primigravida women across levels of partner support
Source: Sci Rep. 2025 Aug 5;15:28541. doi: 10.1038/s41598-025-13586-z (PMC12325668; doi:10.1038/s41598-025-13586-z)
Supplement: Supplementary file 1 — Supplementary Material 1 [file 41598_2025_13586_MOESM1_ESM.docx]

**Supplementary information**


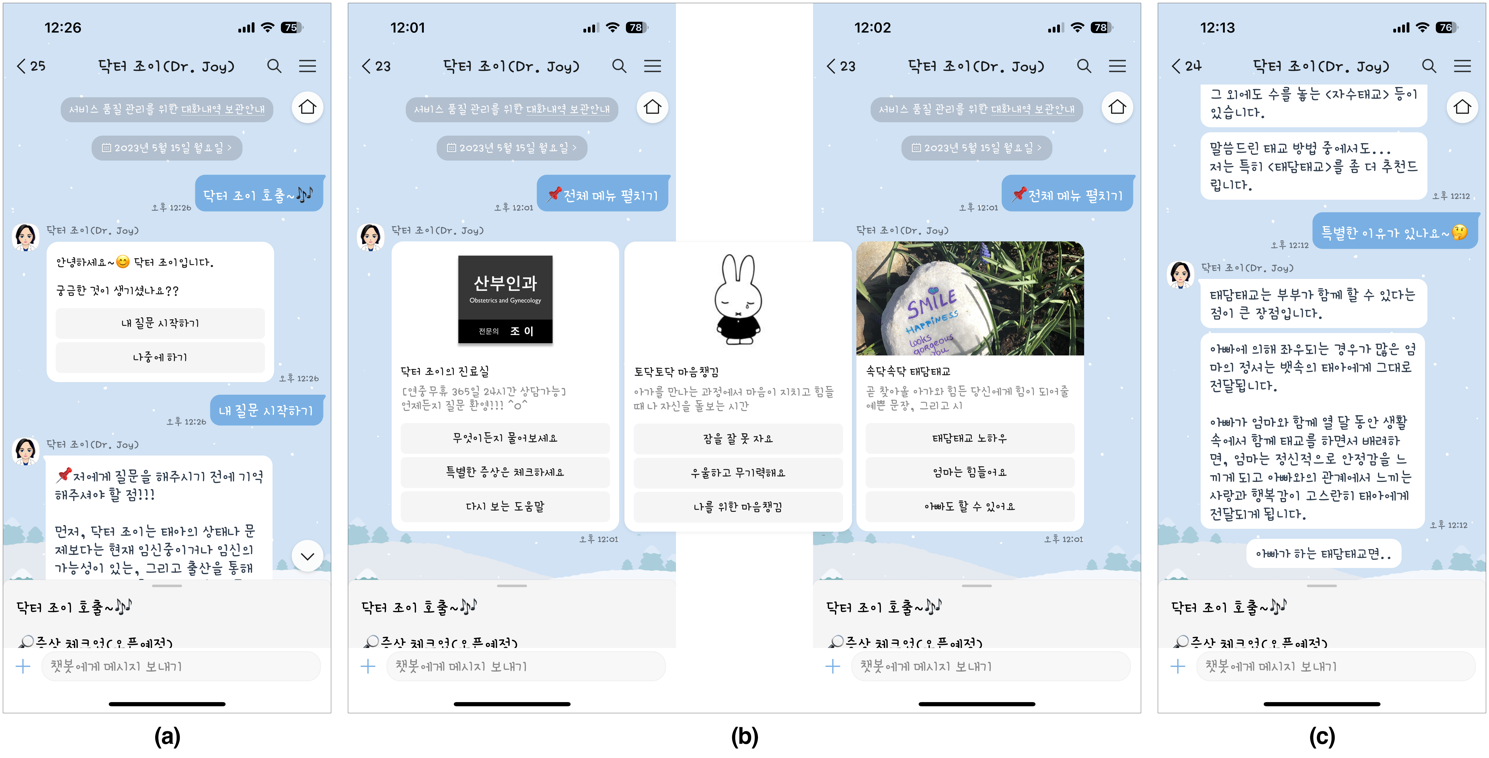


**Supplementary Figure S1.** The original Korean version of Fig. 1 translated into English.


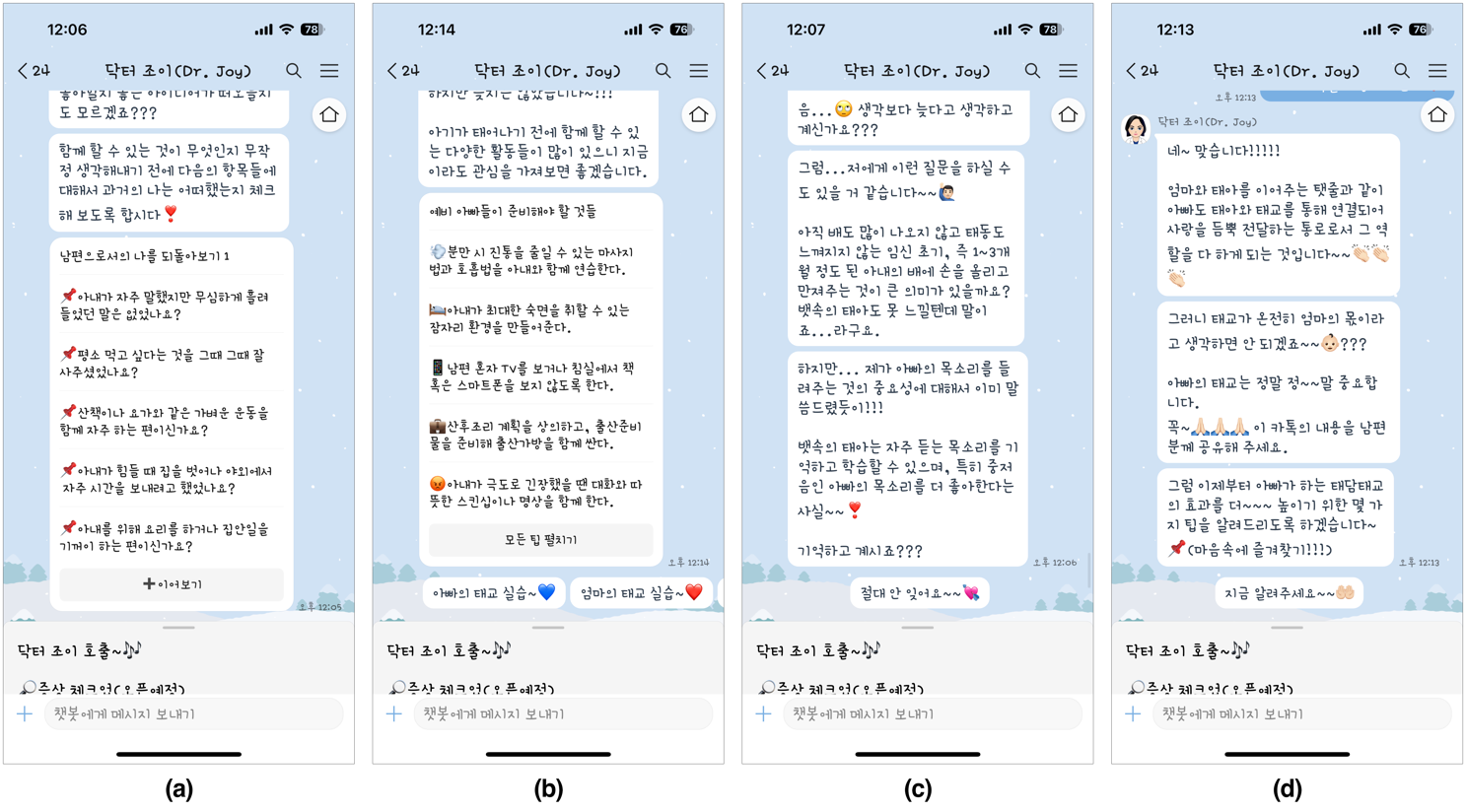


**Supplementary Figure S2.** The original Korean version of Fig. 2 translated into English.

| **Supplementary Data S1.** Illustrative quotes from research participants’ open-ended responses to the most impressive task completed during mobile chatbot-delivered prenatal education. |
| --- |
| ***Cognitive-Behavioral Maternal-Fetal Attachment***   - (1) Maybe, it was good for me and for the fetus, so the fetal movement was very active! [P02, Female, 33 years old] - (2) When I got a 4D ultrasound at 28 weeks, I was so proud to hear from the doctor that the baby seemed to be full of emotion…maybe…because of this prenatal education.   [P11, Female, 32 years old]   - (3) After doing the father-led ‘Taedam’ (i.e., a baby talk called in Korean) at a certain time, I felt the baby became calm as if it recognized his voice or the fetal movement as if in answer to the voice.   [P09, Female, 31 years old]   - (4) When my husband told me a poem every day, I felt the fetus move as if it were responding to his voice.   [P15, Female, 32 years old]   - (5) My husband said he felt the importance of his voice in ‘Taegyo’ (i.e., prenatal education called in Korean). I really saw the baby respond more to his voice than to mine.   [P05, Female, 32 years old]   - (6) While participating in the study, I was able to feel the fetal movement a little more than usual. My husband also didn’t feel much about the presence of the fetus in my womb, but through the task completion, he felt its presence and quickening a little more. As he felt the existence of the baby, there was a change that paid more attention to me who was pregnant. Not too long ago, we thought ‘Taedam’ was nothing special, but through this program, the preparation and mindset for becoming a parent changed, and it was an opportunity to interact with baby a little more.   [P32, Female, 31 years old]  ***Affective Maternal-Fetal Attachment***   - (7) While doing ‘Taedam’ with my husband, I could feel that bonding among us became more solid, and we do ‘Taedam-taegyo’ every morning and evening like a habit.   [P33, Female, 31 years old]   - (8) Even though we didn’t have a set schedule, my husband and I always spent time reading to our child after dinner. It was so nice.   [P43, Female, 30 years old]   - (9) I usually asked my husband to read a book and wanted him to spend more time with me. He didn’t change much when I talked to him directly. As my husband also saw the information on the chatbot, he also came to understand prenatal education more. Thanks to the chatbot, I became happy because he talked more with the baby and the time I spent with him increased.   [P14, Female, 37 years old]   - (10) Every morning when we woke up and every night before we went to bed, my husband called the baby’s nickname with his voice and greeted the baby with a pat on the belly. Every time he did that, I felt happy as a mother and the baby felt comfortable.   [P34, Female, 31 years old]   - (11) While following Dr. Joy’s instructions, the prenatal education app presented poems to us, so the time to practice ‘Taegyo’ with my husband became longer and less burdensome. As a result, the frequency of taegyo increased. In the past, we usually did taegyo in the evening, but now it was good that we could easily start it by turning on the app whenever we had time.   [P64, Female, 29 years old]   - (12) I thought that a mother played a leading role in ‘Taegyo’ and a father only played a supporting role…, but through Dr. Joy’s prenatal classes, I realized that the importance of father-inclusive prenatal education. At first, my husband felt somewhat awkward…, but now he has more time with the babies in my womb, saying, ‘I’ll read you a good phrase.’ I also get to have time to think about my twins.   [P07, Female, 34 years old]   - (13) Since this is my first pregnancy, I thought that ‘Taegyo’ would be proper education only if I received formal education such as prenatal classes or prepared it systematically, but it was so good to be able to assess it easily, comfortably, and daily via KakaoTalk. Particularly, before that, my husband found it difficult to do taegyo because he seemed to believe that it is taegyo to make time for taegyo entirely.   [P35, Female, 36 years old]   - (14) I was looking for some fairy tales or poems to read to my child. It was especially nice to have my husband participate in prenatal education, rather than reading to the baby by myself. For me, participating in the prenatal education program was an opportunity to share information with my husband and study together.   [P04, Female, 38 years old]  ***Impressions on Mobile Chatbot-Based Prenatal Education Program***   - (15) In ‘Taegyo’ with my husband, we often read fairy tale books, but when my husband was tired, he sometimes did not do it at all or finish it early.   [P64, Female, 29 years old]   - (16) Before that, I was a little lazy and annoyed, but it was good that I was able to get information from time to time through the chatbot, learn about ‘Taegyo’, promise myself to become a better father, and put it into practice.   [P11, Male (P11’s spouse), 32 years old]   - (17) As I am pregnant and still working, I always felt nervous and my body felt tense, but through ‘Taegyo’, my body relaxed and abdominal tightening eased. Most of all, I felt good because my mind was stable, and it was also good to be able to feel a sense of happiness. I think the time spent having ‘Taedam’ time with my husband was the best.   [P15, Female, 32 years old]   - (18) Through the ‘Taedam-taegyo’ program, I got to know the father’s and mother’s heart, and even the heart of the fetus in my womb, and I felt so good and happy because we became more intimate and more lovely family than before participating in this program.   [P02, Female, 33 years old]   - (19) Overall, I was satisfied with the chatbot’s utterance that sympathized with depression that could be caused by changes in women before and after pregnancy.   [P41, Female, 31 years old] |
